# Major adverse cardiac events in patients with amiodarone-induced thyrotoxicosis undergoing thyroidectomy: a multicenter study

**DOI:** 10.1530/ETJ-25-0292

**Published:** 2026-04-13

**Authors:** Samuel Frey, Christelle Volteau, Hélène Lasolle, Cécile Caillard, Delphine Drui, Sabine Pattier, Françoise Borson-Chazot, Laure Maillard, Jean-Christophe Lifante, Eric Mirallié

**Affiliations:** ^1^ Nantes Université, CHU Nantes, Chirurgie Cancérologique, Digestive et Endocrinienne, Institut des Maladies de l’Appareil Digestif, Nantes, France; ^2^ Nantes Université, CHU Nantes, CNRS, INSERM, l’institut du thorax, Nantes, France; ^3^ Nantes Université, CHU Nantes, DRCI, Département Promotion, Nantes, France; ^4^ Service d’endocrinologie, Hôpital Louis-Pradel, Hospices civils de Lyon, Lyon, France; ^5^ Nantes Université, CHU Nantes, Service d’Endocrinologie, Diabétologie et Nutrition, l’institut du thorax, Nantes, France; ^6^ Nantes Université, CHU Nantes, Service de cardiologie, Hôpital Nord Laennec, Nantes, France; ^7^ Service de chirurgie endocrinienne, Hôpital Lyon Sud, Hospices civils de Lyon, Pierre-Bénite, France

**Keywords:** thyoidectomy, amiodarone, thyrotoxicosis, cardiovascular risk

## Abstract

**Objective:**

Amiodarone-induced thyrotoxicosis (AIT) affects 5–10% of patients on amiodarone therapy, increasing cardiovascular (CV) risk. Total thyroidectomy is proposed for refractory cases, but its long-term impact on major adverse cardiovascular events (MACEs) remains uncertain. This study evaluates post-operative MACEs and cardiac mortality in AIT patients with and without pre-operative left ventricular ejection fraction (LVEF) impairment.

**Methods:**

Patients undergoing total thyroidectomy for AIT from 2010 to 2023 in two French referral centers were retrospectively included. They were divided into two groups based on pre-operative LVEF (<40% vs ≥40%). The main outcomes were the post-operative MACEs and cardiac mortality at one and five years.

**Results:**

Among 101 patients, 26 had LVEF < 40% (group 1), while 75 had LVEF ≥ 40% (group 2). Patients in group 1 were younger, had higher ASA (American Society of Anesthesia) scores, and had more severe cardiac conditions. The median duration of exposure to AIT was 2.7 (Q1; Q3 1.3; 4.7) months. Overall, perioperative mortality was 2.0%. Group 1 showed higher 5-year MACE occurrence (61.4 vs 24.0%, *P* = 0.0005) and higher cardiac mortality (30.8 vs 1.3%, *P* = 0.0001). Multivariate regression identified younger age at AIT diagnosis and baseline LVEF < 40% as significantly associated with a higher 5-year MACE rate. Among patients with baseline LVEF < 40%, those with early post-operative LVEF improvement displayed a lower rate of 5-year MACEs (47.1 vs 100%, *P* = 0.022).

**Conclusion:**

Patients with LVEF < 40% carries a high long-term CV risk despite early thyroidectomy. Post-operative LVEF assessment may help identify high-risk patients. In contrast, those with LVEF ≥ 40% have a favorable prognosis.

## Introduction

Amiodarone treatment induces thyroid disorders, including hypothyroidism and thyrotoxicosis, in up to 20% of patients under treatments (1, 2), resulting from its high iodine content (3, 4). Among them, amiodarone-induced thyrotoxicosis (AIT), observed in 5–10% of patients in Europe (1), is of particular concern because of its associated elevated cardiovascular (CV) risk in patients with already fragile heart condition. Indeed, compared with other causes of thyrotoxicosis, AIT has been associated with a higher risk of CV events and mortality (5).

AIT initial medical management is of particular importance because it impacts the risk of CV events (6). The modality depends on the type of AIT, defined by its physiopathology. Type 1 AIT, resulting from iodine overload leading to abnormal thyroid hormone synthesis by a thyroid with underlying abnormalities, may respond to high doses of antithyroid drug (ATD) therapy. Type 2 AIT, resulting from thyrocyte destruction, should be treated using corticosteroids (1, 7). An intermediate form, mixed or indefinite, resulting from both mechanisms, has also been described (8). Both ATD and corticosteroids are used for the latter, or in case of resistance to medical therapy (9) (absence of biochemical improvement within 4–6 weeks (8)).

However, optimal medical therapy may be insufficient, as only 36 and 58% of AIT type 1 and 2 respond to ATD and corticosteroids, respectively (10). In persistent AIT, total thyroidectomy has been proposed as the treatment of choice (8), with comparable morbidity with total thyroidectomy for other indications (11). Nevertheless, small cohorts have suggested that these patients remain at risk of high CV mortality (11, 12). In particular, impaired left ventricular ejection fraction (LVEF) has been described as one of the main factors associated with CV mortality in patients with AIT (5, 11, 13). While total thyroidectomy has been shown to improve post-operative LVEF (11, 14), the impact of this benefit on the CV risk remains unclear. Moreover, beyond CV mortality, post-operative major CV events (MACEs) have not been studied in these patients after total thyroidectomy.

The aim of the present study was to assess the post-operative rate of MACEs and cardiac mortality in AIT patients with and without initial LVEF impairment in a large cohort of patients operated for AIT in two French referral centers and to determine pre-operative factors associated with these events.

## Materials and methods

### Patients and data collection

In this retrospective study, patients who underwent total thyroidectomy for ongoing AIT in two referral centers (Lyon Sud University Hospital and Nantes University Hospital) between January 2010 and July 2023 with available pre-operative LVEF evaluation were included. Exclusion criteria were age under 18, pregnancy, adults under guardianship, lobectomy or sub-total thyroidectomy, and preventive thyroidectomy before amiodarone reintroduction for ancient AIT with successful medical management (suspension of medical treatment >1 year). Patients with unavailable pre-operative data concerning LVEF status (≥40% or <40%) or unavailable post-operative follow-up data were also excluded. All data were retrieved from medical records.

Patients were divided into two groups based on pre-operative LVEF: <40% (group 1) and ≥40% (group 2).

Consent was based on the non-opposition principle. This protocol has been validated by the Local Ethics Committee (*Groupe Nantais d’Ethique dans le Domaine de la Santé* (GNEDS), number 24-23-02-274).

### Definition of AIT

AIT was defined, in patients under amiodarone therapy, by a suppressed serum TSH level with elevated serum free T3 and/or T4 levels. AIT subtypes were identified in accordance with the European Thyroid Association (ETA) guidelines (8): type 1 AIT was defined by the presence of diffuse/multinodular goiter, positive TSH receptor antibodies, and/or increased thyroid vascularity on ultrasound color Doppler flow; type 2 AIT was defined by a normal thyroid gland, absence of TSH receptor antibodies and low vascularity on ultrasound color Doppler flow. The absence of radioactive iodine uptake, when performed, also suggested type 2 AIT. When available data were insufficient to distinguish between type 1 and type 2 AIT, ‘undetermined’ AIT was considered. Initial medical management (ATD/corticosteroids) was conducted according to the ETA guidelines (8), with combined treatments and sometimes adjunction of perchlorate in case of persistent thyrotoxicosis.

### Perioperative cardiac evaluation and general evaluation

All patients underwent LVEF measurement by echocardiography before surgery. LVEF impairment was defined by a LVEF < 40% in accordance with the Framingham Heart Study (15). In patients with impaired pre-operative LVEF, post-operative LVEF measurements were retrieved. Underlying cardiopathy was classified according to the 2023 European Society of Cardiology (ESC) guidelines (16) into coronary artery disease (CAD), hypertensive cardiopathy, valvular cardiopathy, congenital heart disease, and cardiomyopathy. Patients’ general condition was evaluated using the Charlson comorbidity index (17) and the American Society of Anesthesia (ASA) score at the time of surgery.

### Surgical indications

Indications for surgery were AIT persistence/dependence requiring medical management, underlying thyroid pathology, or cardiac indication (amiodarone reintroduction, cardiac transplantation, or urgent thyrotoxicosis control) (8).

Total thyroidectomy was performed by four experienced endocrine surgeons via Kocher incision with systematic neuromonitoring. Serum calcium level was measured on day 1. Post-operative medical and surgical complications (cervical hematoma requiring reintervention, hypocalcemia, recurrent laryngeal nerve palsy, and wound infections) were recorded. Post-operative death was defined as death occurring within 30 days after surgery.

### Outcome definitions

Time for surgery referral was defined by the time from the first diagnosis of AIT and thyroidectomy. At the time of surgery, patients were considered euthyroid if both serum free T3 and T4 were within normal ranges. As hormone levels were assessed using different kits across centers, only their normality (per each kit’s reference range) was considered, and raw data are not presented in the present study. Duration of exposure to AIT was defined by the time from first diagnosis of AIT to the first normalization of free thyroid hormones (either under medical therapy or after surgery). Cardiothyreosis was defined by the occurrence of rhythm disorder (including *de novo* or recurrence/aggravation of pre-existent atrial fibrillation), heart failure, or myocardial infarction in patients with thyrotoxicosis. Palpitations without evidence of rhythm disorder on electrocardiography were not considered. MACEs were defined by the occurrence of heart failure requiring hospitalization, myocardial infarction, stroke including transient ischemic attack, ventricular arrhythmias requiring hospital admission, and/or cardiac mortality.

### Statistical analysis

Continuous variables are presented as median (first; third quartiles), and categorical variables as number (percentage). Comparisons were made using the Mann–Whitney test and the chi-squared test or Fisher’s exact test for continuous and categorical variables, respectively. Survival without MACEs and survival without cardiac mortality were estimated using the Kaplan–Meier method and compared with log-rank tests. Predictive factors of these outcomes were searched with univariate and multivariate logistic regression models. Because of redundant variables (age and cardiopathy), the Charlson comorbidity index was excluded from the multivariate model. All statistical tests were two-sided at the 5% level of significance. No imputations were done on missing data. Statistical analyses were conducted using SAS software, version 9.4 (SAS Institute Inc., USA).

## Results

### Patients’ baseline characteristics

**Figure 1:**
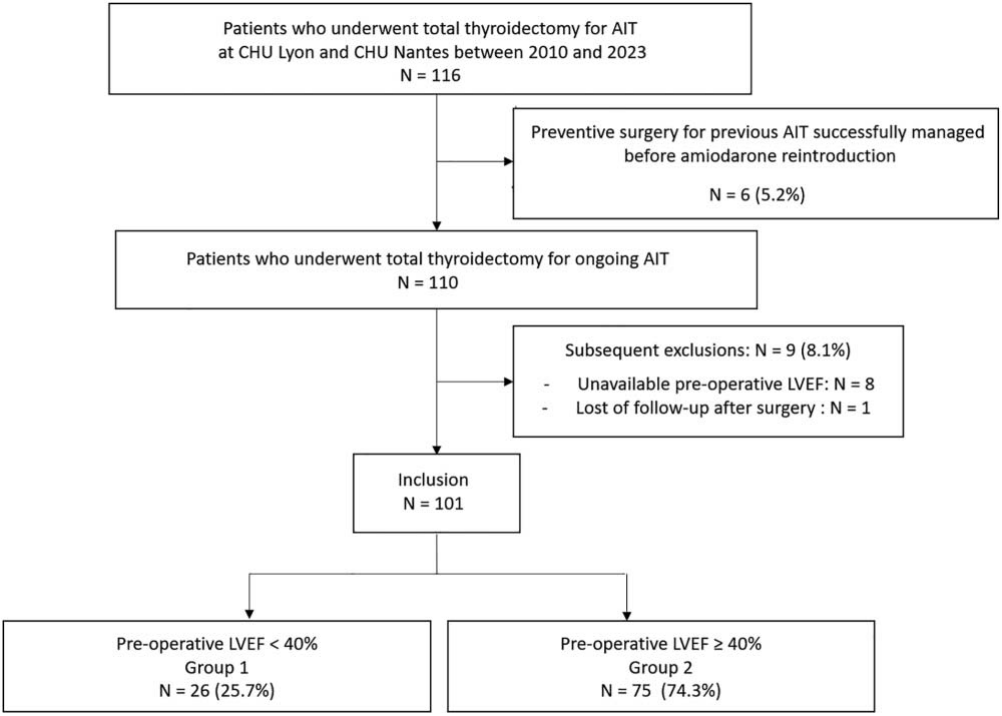
Flow chart. AIT, amiodarone induced thyrotoxicosis; LVEF, left ventricular ejection fraction.

Between 2010 and 2023, a total of 116 patients underwent total thyroidectomy for AIT at Lyon Sud and Nantes University Hospitals. Among them, six (5.2%) were excluded because surgery was conducted in order to reintroduce amiodarone after a resolved AIT episode. Nine patients (8.1%) were subsequently excluded owing to unavailable pre-operative LVEF measurement or loss to follow-up after surgery (see flow chart; Fig. 1). One hundred and one patients were therefore included: 26 (25.7%) had a pre-operative LVEF < 40% and belonged to group 1, while 75 (74.3%) had a pre-operative LVEF ≥ 40% and belonged to group 2.

Group 1 patients were significantly younger (58.5 (48.0; 66.3) vs 70.8 (58.1; 78.1) years, *P* = 0.0014), had a higher ASA score (all ≥ 3), had more cardiopathy (100 vs 65.3%, *P* = 0.0002), and comprised a higher proportion of patients with an implantable cardioverter defibrillator (61.5 vs 13.3%, *P* < 0.0001) than group 2 patients (Table 1). The median LVEF in group 1 was 27.0% (20.0; 35.0). Otherwise, the medical reason to introduce amiodarone therapy, the rate of patients who displayed cardiothyreosis, and the repartition between AIT types did not differ between groups. The rate of euthyroid patients at the time of surgery showed a trend toward a lower rate in group 1 patients (15.4 vs 26.7% for groups 1 and 2, respectively). The median time for surgery referral (2.4 (1.4; 4.9) and 3.2 (1.8; 7.7) in groups 1 and 2, respectively) and duration of exposure to AIT (2.4 (1.1; 4.8) and 2.8 (1.3; 4.5)) were also comparable between groups.

**Table 1:** Population characteristics. Continuous data are expressed as median (first; third quartiles), and categorical variables as number (percentage). Comparisons were made with the Mann–Whitney test for continuous variables and Fisher’s exact test or the chi-square test for categorical variables. Statistically significant values (*P <* 0.05) are presented in bold.

	Overall	Group 1: LVEF < 40%	Group 2: LVEF ≥ 40%	*P*-value
*n*	101	26	75	
Age, years	66.0 (55.5; 77.0)	58.5 (48.0; 66.3)	70.8 (58.1; 78.1)	**0.0014**
Sex ratio (male/female)	76/25 (72.2/24.8)	23/3 (88.5/11.5)	53/22 (70.7/29.3)	0.07
BMI, Kg/m^2^	26.0 (24.0; 31.2)	26.8 (24.0; 31.4)	26.0 (23.7; 31.2)	0.68
Charlson index	3.0 (2.0; 4.0)	2.0 (1.0; 4.0)	3.0 (2.0; 5.0)	0.055
ASA				**<0.0001**
1	0	0	0	
2	25 (24.7)	0	25 (33.3)	
3	58 (57.4)	16 (61.5)	42 (56.0)	
4	18 (17.8)	10 (38.5)	8 (10.6)	
5	0	0	0	
Cardiopathy	75 (74.3)	26 (100)	49 (65.3)	**0.0002**
CAD	20 (19.8)	8 (30.8)	12 (16.0)	0.011*
Congenital cardiopathy	37 (36.6)	14 (53.8)	23 (30.7)	
Valvular cardiopathy	5 (5.0)	1 (1.0)	4 (5.3)	
Hypertensive cardiopathy	3 (3.0)	0	3 (4.0)	
Cardiomyopathy	4 (4.0)	2 (2.0)	2 (2.7)	
Mixed	6 (5.9)	1 (1.0)	5 (6.7)	
ICD carrier	26 (25.7)	16 (61.5)	10 (13.3)	**<0.0001**
Median pre-operative LVEF, %	50.0 (35.0; 60.0)	27.0 (20.0; 35.0)	60.0 (50.0; 65.0)	**<0.0001**
Cardiothyreosis	42 (41.6)	13 (50.0)	29 (38.7)	0.31
Indication for amiodarone therapy				0.77
Supraventricular arrhythmia	84 (84.0)	21 (80.8)	63 (85.1)	
Ventricular arrhythmia	16 (16.0)	5 (19.2)	11 (14.9)	
AIT type				1.00
1	18 (17.8)	4 (15.4)	14 (18.7)	
2 or undetermined	83 (82.18%)	22 (84.62%)	61 (81.33%)	
Time for surgery referral, months	3.0 (1.6; 7.1)	2.4 (1.4; 4.9)	3.2 (1.8; 7.7)	0.30
Euthyroidism at surgery	24 (23.8)	4 (15.4)	20 (26.7)	0.24
Serum free T3 normalization at surgery	47 (50.5)	13 (52.0)	34 (50.0)	0.86
Duration of exposure to AIT, months	2.7 (1.3; 4.7)	2.4 (1.1; 4.8)	2.8 (1.3; 4.5)	0.59
Median follow-up after diagnosis, months	54.1 (25.7; 91.0)	60.2 (24.3; 91.4)	49.9 (25.7; 91.0)	0.58
1-year MACEs	17 (16.8)	9 (34.6)	8 (10.7)	**0.012**
5-year MACEs	34 (33.7)	16 (61.5)	18 (24.0)	**0.0005**
1-year cardiac mortality	1 (1.0)	1 (3.9)	0	0.26
5-year cardiac mortality	9 (8.9)	8 (30.8)	1 (1.3)	**0.0001**

* Comparing all cardiovascular conditions.

LVEF, left ventricular ejection fraction; AIT, amiodarone-induced thyrotoxicosis; BMI, body mass index; ASA, American Society of Anesthesiologists; CAD, coronary artery disease; ICD, implantable cardioverter-defibrillator; T3, triiodothyronine; MACEs, major adverse cardiovascular events.

### AIT management according to the pre-operative LVEF

The rates of patients treated with corticosteroids (80.8% in group 1 and 77.3% in group 2) and ATD (80.8 and 86.7%, Supplemental Table 1 (see section on Supplementary materials given at the end of the article)) were comparable between groups. The majority of patients (69.2% in group 1 and 66.6% in group 2) were treated with both corticosteroids and ATD. A significantly higher proportion of group 1 patients was treated with sodium perchlorate (42.3 vs 17.3%, *P* = 0.001) and plasmapheresis (19.2 vs 5.3%, *P* = 0.047).

Early post-operative medical complications were significantly more frequent in group 1 patients (Table 2) with 14.9 and 10.9% of patients who displayed medical and surgical post-operative complications, respectively. Hematoma requiring reintervention, post-operative hypocalcemia, and recurrent laryngeal nerve palsy occurred in 2, 5 (persistent in 2), and 1 patient, respectively. Two patients (one per group) died <30 days after surgery, one from infectious pneumonia, and one from cellulitis resulting in septic shock.

**Table 2:** Surgery characteristics and post-operative course in AIT patients. Continuous data are expressed as median (first; third quartiles), and categorical variables as number (percentage). Comparisons were made with the Mann–Whitney test for continuous variables and Fisher’s exact test for categorical variables. *P <* 0.05 was considered statistically significant.

	Overall	Group 1: LVEF < 40%	Group 2: LVEF ≥ 40%	*P*-value
*n*	101	26	75	
Operative time, minutes	90 (75.3; 107.3)	95 (77.0; 123.5)	90.0 (74.5; 102.5)	0.18
Medical complications^†^	15 (14.9)	6 (23.1)	9 (12.0)	0.04
Surgical complications	11 (10.9)	4 (15.4)	7 (9.3)	0.27
Hematoma requiring reintervention	2 (2.0)	2 (7.7)	0	-
Hypocalcemia	5 (5.0)	5 (19.2)	0	-
Recurrent laryngeal nerve palsy	1 (1.0)	0	1 (1.3)	-
Wound infection	2 (2.0)	2 (7.7)	0	-
Others*	1 (1.0)	1 (3.8)	0	-
Mortality < 30 days	2 (2.0)	1 (3.8)	1 (1.3)	1.00

* Other surgical complications included one patient with hematoma not requiring reintervention.

^†^ Medical complications included three acute cardiac failures, two rapid atrial fibrillations, one fall resulting in a thigh hematoma with deglobulization, one cellulitis resulting in septic shock, two hypotensive episodes, one myocardial infarction associated with infectious pneumonia, and one urinary tract infection.

### Post-operative cardiovascular outcomes according to the study group

**Figure 2:**
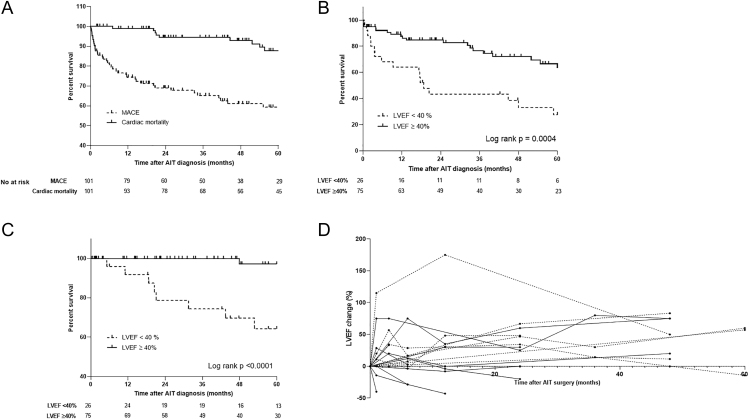
Post-operative MACEs and cardiac mortality according to pre-operative LVEF status, and post-operative LVEF change (% of pre-operative LVEF) in those with impaired pre-operative LVEF. (A) MACE (dashed line)- and cardiac mortality (solid line)-free survival estimation in all patients; (B) MACE- and (C) cardiac mortality-free survival in patients with LVEF < 40% and patients with LVEF ≥ 40%. (D) Post-operative LVEF change (% of pre-operative LVEF) in those with impaired pre-operative LVEF (solid line: patients with MACE occurrence within 5 years, dashed line: patients without MACE occurrence within 5 years). MACEs: major adverse cardiovascular events.

The median post-operative follow-up after AIT diagnosis was 60.2 (24.3; 91.4) months in group 1 and 49.9 (25.7–91.0) months in group 2. Overall, 17/101 patients (16.8%) displayed MACEs and 1/101 patient (1.0%) died from cardiac cause during the first year after AIT diagnosis, while 34/101 (33.7%) and 9/101 (8.9%) displayed MACEs and cardiac death, respectively, during the five-year follow-up (Table 1, Fig. 2A). MACEs at 1 and 5 years were significantly more frequent in group 1 patients (Fig. 2B) (34.6 vs 10.7% at 1 year, *P* = 0.012; and 61.4 vs 24.0% at 5 years, *P* = 0.0005, Table 1). The 5-year cardiac mortality was accordingly more frequent in group 1 patients (Fig. 2C) (30.8 vs 1.3%, *P* = 0.0001, Table 1).

Factors associated with 1- and 5-year MACEs and 1-year cardiac mortality were studied. In univariate analysis, only the pre-operative LVEF < 40% was significantly associated with 1-year MACEs, while none of the variables were identified in multivariate analysis (data not shown). A younger age at AIT diagnosis, a pre-operative LVEF < 40%, a shorter time of exposure to AIT, a shorter time for surgery referral, and the absence of euthyroidism at the time of surgery were significantly associated with 5-year MACE occurrence in univariate analysis (Table 3). Multivariate analysis identified that younger age at AIT diagnosis and pre-operative LVEF < 40% were the only variables significantly associated with 5-year MACE occurrence (odds ratios 0.96 (95% CI: 0.91; 1.00), and 3.81 (1.19; 12.20), respectively). When analyzed as a continuous variable, pre-operative LVEF was the only variable associated with 5-year MACE occurrence in multivariate analysis (Supplemental Table 2). Accordingly, the lower pre-operative LVEF was the only variable associated with 5-year cardiac mortality in univariate analysis (data not shown).

**Table 3:** Univariate and multivariate logistic regression analysis of factors associated with the occurrence of post-operative major cardiovascular events in patients operated for AIT. Statistically significant values (*P* < 0.05) are in bold.

Variable	Univariate analysis		Multivariate analysis	
	OR (95% CI)	*P*-value	OR (95% CI)	*P*-value
Age at AIT diagnosis	0.96 (0.93; 0.99)	**0.016**	0.96 (0.91; 1.00)	**0.04**
Female sex	0.71 (0.26; 1.90)	0.49	1.49 (0.43; 5.25)	0.53
BMI	0.98 (0.92; 1.04)	0.46	0.97 (0.90; 1.05)	0.48
Cardiopathy	2.65 (0.90; 7.80)	0.077	1.08 (0.29; 4.09)	0.90
Charlson index	0.84 (0.66; 1.07)	0.16	-	-
Cardiothyreosis	2.02 (0.87; 4.66)	0.10	1.67 (0.58; 4.82)	0.34
Patients with pre-operative LVEF < 40%	5.07 (1.96; 13.12)	**0.0008**	3.81 (1.19; 12.20)	**0.024**
Duration of exposure to AIT	0.82 (0.69; 0.98)	**0.028**	0.82 (0.63; 1.08)	0.16
Euthyroidism at surgery	0.21 (0.06; 0.77)	**0.019**	0.41 (0.07; 2.46)	0.33
Time for surgery referral	0.85 (0.74; 0.98)	**0.021**	0.99 (0.82; 1.20)	0.93

OR, odds ratio; 95% CI, 95% confidence interval; BMI, body mass index; LVEF, left ventricular ejection fraction; AIT, amiodarone-induced thyrotoxicosis.

### Post-operative LVEF change in patients with impaired pre-operative LVEF

The post-operative LVEF change during the five-year follow-up in patients with an impaired pre-operative LVEF (group 1 patients) is described in Fig. 2D. Post-operative LVEF measurement was missing in two patients with an impaired pre-operative LVEF. Among 24 patients with pre- and post-operative LVEF measurements, 17 showed improvement, while 7 had a stable/worsened LVEF at the first measurement (median 2.6 months (1.46; 9.73)). The baseline characteristics, including the median pre-operative LVEF, were comparable between these two subgroups, except for a higher ASA score in the stable/worsened group (*P* = 0.0088, Table 4). Patients with an improved LVEF had longer AIT exposure (3.0 (1.6; 4.8) vs 1.1 (0.4; 2.5) months, *P* = 0.032). Notably, all patients with a stable/worsened post-operative LVEF displayed 5-year MACEs, versus 8/17 patients with an improved post-operative LVEF (47.1%, *P* = 0.022). No significant difference was observed between groups regarding 1- and 5-year cardiac mortality.

**Table 4:** Characteristics of patients with improved or stable/worsened post-operative LVEF at first measurement (among those with pre-operative LVEF < 40%). Continuous data are expressed as median (first; third quartiles), and categorical variables as number (percentage). Comparisons were made with Mann–Whitney test for continuous variables and Fisher’s exact test or the chi-square test for categorical variables. Statistically significant values (*P <* 0.05) are presented in bold.

	Improved LVEF	Stable/worsened LVEF	*P*-value
*n*	17	7	
Age at AIT diagnosis, years	58.7 (48.9; 64.5)	66.3 (46.9; 73.4)	0.57
Sex ratio (males/females)	14/3 (82.3/17.7)	7/0 (100/0)	0.53
BMI, Kg/m^2^	28.0 (25.0; 30.0)	24.0 (21.0; 31.4)	0.37
Charlson index	2.0 (1.0; 4.0)	3.0 (1.0; 5.0)	0.85
ASA			**0.0088**
3	13 (76.5)	1 (14.3)	
4	4 (23.5)	6 (85.7)	
Cardiopathy	17 (100)	7 (100)	
CAD	6 (35.3)	2 (28.6)	1.00
Congenital cardiopathy	1 (5.9)	0	
Valvular cardiopathy	1 (5.9)	0	
Cardiomyopathy	8 (47.1)	4 (57.1)	
Mixed	1 (5.9)	1 (14.3)	
ICD carrier	10 (58.8)	5 (71.4)	0.67
Median pre-operative LVEF, %	26.5 (20.0–33.8)	30.0 (22.5–35.0)	0.52
Cardiothyreosis	9 (52.9)	4 (57.1)	1.00
Indication for amiodarone therapy			
Supraventricular arrhythmia	13 (76.5)	6 (85.7)	1.00
Ventricular arrhythmia	4 (23.5)	1 (14.3)	
AIT type			
1	2 (11.8)	1 (14.3)	1.00
2 or undetermined	15 (88.2)	6 (85.7)	
Time for surgery referral, months	3.0 (1.6; 4.9)	1.4 (1.1; 2.5)	0.078
Euthyroidism at surgery	2 (11.8)	1 (14.3)	1.00
Serum free T3 normalization at surgery	9 (52.9)	3 (42.9)	1.00
Duration of exposure to AIT, months	3.0 (1.6; 4.8)	1.1 (0.4; 2.5)	**0.032**
Median follow-up after diagnosis, months	62.1 (44.6; 101.5)	54.1 (20.1; 72.5)	0.35
1-year MACEs	4 (23.5)	4 (57.1)	0.17
5-year MACEs	8 (47.1)	7 (100.0)	**0.022**
1-year cardiac mortality	0	1 (14.3)	0.29
5-year cardiac mortality	3 (17.7)	4 (57.1)	0.13

LVEF, left ventricular ejection fraction; AIT, amiodarone-induced thyrotoxicosis; BMI, body mass index; ASA, American Society of Anesthesiologists; CAD, coronary artery disease; ICD, implantable cardioverter-defibrillator; T3, triiodothyronine; MACEs, major adverse cardiovascular events.

## Discussion

To our knowledge, this retrospective, bi-centric study represents the largest surgical series of AIT patients reported to date. It showed that, in this population operated promptly after the diagnosis and characterized by an elevated post-operative MACE occurrence and cardiac mortality, a pre-operative impaired LVEF was associated with 5-year post-operative MACEs. Overall, surgery was associated with a relatively low short-term perioperative mortality and an acceptable rate of surgical complications. In patients with a pre-operative LVEF < 40%, the absence of early post-operative LVEF improvement could be a predictive factor of 5-year MACE occurrence.

While AIT is known to be associated with a high MACE rate under amiodarone therapy (18), the present study showed that MACEs remained frequent even after thyroidectomy, occurring in 16.8 and 33.7% of patients at 1 and 5 years, respectively. Although this could reflect the underlying cardiac condition rather than AIT itself, Yiu *et al.* reported a higher long-term MACE rate in non-operated AIT patients compared to euthyroid patients with similar cardiac profiles (31.6 vs 10.7%, *P* < 0.01; median follow-up 48 months) (18). Recently, Cappellani *et al.* showed that 74.4% of AIT patients displayed MACEs before obtaining euthyroidism, while optimal therapy could reduce this rate to 4.5% (6). In another study including 84 AIT patients (8 underwent thyroidectomy during follow-up), Conen *et al.* reported a 56% MACE rate with a median follow-up of 50 months (range: 17–78) (13). Although no comparison could be made with non-operated patients in the present study, the lower MACE rate compared to the previously cited studies could suggest a favorable impact of surgery on the long-term cardiovascular outcomes. In this line, it has recently been suggested in a meta-analysis including 12 studies (192,208 patients) that surgery for hyperthyroidism from any cause, compared to ATD treatment and radioactive therapy, was associated with significantly decreased risks of all-cause mortality and cardiovascular mortality (19).

For instance, one of the main identified predictors of CV outcomes in AIT patients is the LVEF (11, 13). This study confirms these results, as patients with an impaired baseline LVEF showed an elevated rate of 1- and 5-year MACE rate, along with an elevated 5-year cardiac mortality rate compared with patients with a pre-operative LVEF ≥ 40%. These results are in accordance with our previous study, in which 47.0% of patients with an impaired LVEF died from cardiac cause 5 years after surgery compared with 2.9% of patients with a normal baseline LVEF (11). Others showed that, in unoperated AIT patients, long-term MACEs occurred in 73 and 49% of patients with an impaired LVEF and a normal LVEF, respectively (*P* = 0.04) (12). By contrast, in our study, patients with a LVEF ≥ 40% had a low cardiac mortality (1.3% at 5 years), suggesting the safety of surgery in these patients. In our study, patients with a LVEF < 40% were significantly younger, and a younger age at AIT diagnosis was associated with 5-year MACE occurrence, which may appear counterintuitive. Although this finding could result from differences between underlying etiologies of cardiopathy (53.8 vs 30.7% had congenital cardiopathies), we cannot exclude an inclusion bias, as older and frail patients may have been less likely addressed to surgery. A survival bias is also possible, with a higher mortality after diagnosis in older patients before surgical referral.

As baseline LVEF is a difficult variable to control, several studies have aimed to identify predictors of post-operative CV outcomes in AIT patients. Previous studies highlighted an association between the duration of exposure to thyrotoxicosis, linked with the time for surgery referral, and the post-operative cardiac mortality. In a study including 64 operated AIT patients, Cappellani *et al.* showed that early surgery (before normalization of thyroid function) was associated with a lower 5-year cardiac mortality in patients with an impaired LVEF and that longer thyrotoxicosis exposure was associated with an increased 5-year cardiac mortality (12). Similarly, in our previous single-center study of 51 patients, a baseline LVEF < 40% and delayed surgery were significantly associated with 5-year cardiac mortality (11). Therefore, a rapid restauration of euthyroidism with surgery in all patients with an impaired LVEF is advised in accordance with the ETA guidelines (8, 20, 21). Nevertheless, the present study did not find a significant association between the time of exposure to AIT and CV outcomes. The most plausible explanation is the fact that patients with AIT in the including centers were likely to be operated promptly after diagnosis while hyperthyroid, especially those with a low LVEF: only 23.8% were operated after restauration of euthyroidism (versus 37.5% in (12) and 47.1% in (11)). Furthermore, the median time of exposure to AIT was inferior to 3 months in the present study, which corresponds to a short exposition, as reported previously (12). This may explain the lack of a significant impact of surgical delay in our cohort, in which patients had already undergone early surgery. The fact that shorter time of exposure to AIT was associated with 5-year MACE rate only in univariate analysis, this association disappearing in multivariate analysis, suggests confounding factors, including the fact that weaker patients may have undergone surgery more promptly.

Prompt restoration of euthyroidism after surgery is associated with early post-operative LVEF improvement (14, 22). Thyrotoxicosis may lead to a reduction in LVEF through excess thyroid hormones, which increase heart rate, reduce myocardial contractile reserve, and increase blood volume (23), especially in elder patients and those with pre-existing cardiac dysfunction. The occurrence of cardiothyreosis (41.6% in our series), especially atrial fibrillation (24), further adds to the cardiovascular burden in these patients. When thyroid hormone excess is prolonged, these mechanisms can lead to adverse cardiac remodeling, ultimately leading to left ventricular fibrosis and dilated heart failure (23). It can be hypothesized that surgery, if performed before irreversible remodeling occurs, may improve cardiac function by promptly restoring euthyroidism in these patients. The present study confirms sustained LVEF improvement in patients with preoperative impairment. Notably, all patients without early LVEF improvement experienced MACEs within five years, suggesting this measure may identify high-risk patients. Despite similar baseline LVEF, those without improvement had higher ASA scores and shorter AIT exposure, suggesting greater severity prompting earlier surgery. These findings highlight the potential prognostic value of early LVEF changes. However, based only on our results, it is not possible to know if this improvement *per se* reduces long-term MACEs, or whether it identifies patients with less severe or reversible cardiac condition. Further studies are needed to identify predictive factors of early post-operative LVEF response after thyroidectomy in AIT patients.

An important result of our study is that surgery in patients with AIT appeared associated with a low short-term perioperative mortality, as well as an acceptable rate of surgical complications. The 30-day post-operative mortality rate of 2.0% observed in our study was lower than that reported in previous series, which described an early post-operative mortality between 5.9 and 26% (11, 12, 25, 26). However, this outcome appears to depend on preoperative LVEF and/or the duration of thyrotoxicosis. In the study of Cappellani *et al.*, patients with a LVEF ≥ 40% had a lower early post-operative mortality (4.0–6.3%), whereas patients with a LVEF < 40% had a low mortality only when surgery was performed promptly after diagnosis (12). This is consistent with our findings in a cohort of patients who underwent early surgery. Therefore, concerns about excessive perioperative mortality in thyrotoxic patients with severe cardiac disease should not, in itself, justify delaying surgery. While a high rate of medical complications is unsurprising in this population, surgical complication rates were comparable to those reported for thyroidectomy performed for other indications (27, 28, 29).

The present study presents several limitations. The first is its retrospective nature, which led to difficulties to establish the classification of AIT into subtypes. We chose to class debatable cases as ‘undetermined’. The second is the lack of control group composed of non-operated AIT patients. Therefore, it is only possible to describe the post-operative outcomes in these patients. It is also not possible, based on the present study, to discuss the surgical indications. The fourth is the fact that the cardiac mortality in the overall cohort in the present study (8.9%) is lower compared to others (12) and our own precedent series (17.6%) (11). Explanations could include i) the bi-centric design of the present study, reflecting variability of this outcome according to the local multidisciplinary management; ii) the fact that patients with AIT were massively addressed to our center since the precedent study, including those with better prognosis who were previously managed only with medical treatment; and iii) the fact that patients were operated shortly after AIT diagnosis according to the recent recommendations. This advocates for future larger multicenter studies. Finally, our study design does not allow us to assess the specific role of thyrotoxicosis *per se* in increasing the risk of MACEs among AIT patients with impaired cardiac status at baseline, as this would have required a comparison with patients with similar cardiac profiles. This approach was adopted by Yu *et al.*, who reported a 2.7-fold higher risk of MACEs in AIT patients compared with patients with comparable cardiac characteristics (18).

## Conclusion

In this retrospective, bi-center study including AIT patients who underwent prompt surgery after diagnosis, impaired baseline LVEF appeared as the strongest predictor of long-term MACEs and cardiac mortality. Overall, perioperative mortality was low, and surgical complication rate was comparable with thyroidectomy performed for other indications, supporting the safety of surgical management for AIT. In patients with preserved LVEF, these results suggest that surgery presents a reliable treatment option for AIT. In patients with impaired cardiac function, surgery was associated with a sustained LVEF improvement in a significant proportion of patients, suggesting the deleterious effect of thyrotoxicosis on heart function. While these patients remained at high CV risk in the long-term after surgery, the early post-operative LVEF measurement may help identify those who remain at increased CV risk.

## Supplementary materials



## Declaration of interest

The authors declare that there is no conflict of interest that could be perceived as prejudicing the impartiality of the work reported.

## Funding

This work did not receive any specific grant from any funding agency in the public, commercial, or not-for-profit sector.

## Author contribution statement

SF conceived and designed the study, acquired and analyzed the data, and drafted the manuscript. CV designed the study, performed analysis, and critically reviewed the manuscript for important intellectual content. HL, SP, and FB-C designed the study and critically reviewed the manuscript for important intellectual content. CC and LM conceived the study and critically reviewed the manuscript for important intellectual content. DD, J-CL, and EM conceived and designed the study and critically reviewed the manuscript for important intellectual content.
