# Supplementary material for: Major adverse cardiac events in patients with amiodarone-induced thyrotoxicosis undergoing thyroidectomy: a multicenter study
Source: Eur Thyroid J. 2026 Apr 13;15(2):ETJ250292. doi: 10.1530/ETJ-25-0292 (PMC13097270; doi:10.1530/ETJ-25-0292)
Supplement: Supplementary file 1 [file supplementary_materials.pdf]

Supplemental Table 1. AIT pre-operative medical management

	**Overall**<br>**N = 101**	**Group 1**<br>**LVEF <40%**<br>**N = 26**	**Group 2**<br>**LVEF ≥40%**<br>**N = 75**	**p-value**
**Corticosteroids**	79 (78.2)	21 (80.8)	58 (77.3)	0.71
Median daily dose, mg	60.0 [40.0; 70.0]	60.0 [50.0; 80.0]	60.0 [40.0; 70.0]	0.12
**ATD**	86 (85.2)	21 (80.8)	65 (86.7)	0.52
**Carbimazol**	49 (48.5)	13 (50.0)	36 (48.0)	0.86
Median daily dose, mg	50.0 [40.0; 60.0]	40.0 [20.0; 60.0]	55.0 [40.0; 60.0]	0.70
**Methimazol**	6 (5.9)	1 (3.9)	5 (6.7)	1.00
Median daily dose, mg	30.0 [20.0; 40.0]	40.0 [40.0; 40.0]	25.0 [20.0; 32.5]	-
**Propylthiouracil**	41 (40.6)	9 (34.6)	32 (42.7)	0.47
Median daily dose, mg	300.0 [200.0; 600.0]	300.0 [300.0; 450.0]	350.0 [175.0; 600.0]	0.95
**Corticosteroids + ATD**	68 (67.3)	18 (69.2)	50 (66.6)	1.00
**Perchlorate**				
Potassium	10 (9.9)	2 (7.7)	8 (10.7)	1.00
Sodium	24 (23.8)	11 (42.3)	13 (17.3)	**0.001**
**Plasmapheresis**	9 (8.9)	5 (19.2)	4 (5.3)	**0.047**

Continuous data are expressed as median [first – third quartile], and categorical variables as number (percentage). Comparisons were done with Mann-Whitney test for continuous variables, and Fisher's exact test for categorical variables. P<0.05 (bold) was considered statistically significant.

Median daily doses are given in mg per day.

ATD: Antithyroid drugs

Supplemental Table 2. Uni- and multivariate logistic regression analysis of factors associated with the occurrence of post-operative major cardiovascular events in patients operated for AIT

	Univariate analysis			
**Variable**	**OR**	**95%CI**	**p-value**	**OR**
**Age at AIT diagnosis**	0.96	[0.93; 0.99]	**0.016**	0.9[illegible]
**Female sex**	0.71	[0.26; 1.90]	0.49	1.6[illegible]
**BMI**	0.98	[0.92; 1.04]	0.46	0.9[illegible]
**Cardiopathy**	2.65	[0.90; 7.80]	0.077	0.2[illegible]
**Charlson index**	0.84	[0.66; 1.07]	0.16	-
**Cardiothyreosis**	2.02	[0.87; 4.66]	0.10	1.6[illegible]
**LVEF (%)**	0.92	[0.89; 0.96]	**<0.0001**	0.9[illegible]
**Duration of exposure to AIT**	0.82	[0.69; 0.98]	**0.028**	0.8[illegible]
**Euthyroidism at surgery**	0.21	[0.06; 0.77]	**0.019**	0.4[illegible]
**Time for surgery referral**	0.85	[0.74; 0.98]	**0.021**	1.0[illegible]

P<0.05 (bold) was considered statistically significant.

OR: Odds ratio, **95%CI:** 95% confidence interval, **BMI:** Body mass index, **LVEF:** Left ventricular ejection fraction, **AIT:** Amiodarone-induced thyrotoxicosis.
